# An electric generator using living *Torpedo* electric organs controlled by fluid pressure-based alternative nervous systems

**DOI:** 10.1038/srep25899

**Published:** 2016-05-31

**Authors:** Yo Tanaka, Shun-ichi Funano, Yohei Nishizawa, Norihiro Kamamichi, Masahiro Nishinaka, Takehiko Kitamori

**Affiliations:** 1 Quantitative Biology Center (QBiC), RIKEN, 1-3 Yamadaoka, Suita, Osaka 565-0871, Japan; 2 Department of Robotics and Mechatronics, Tokyo Denki University, 5 Senju-asahi-cho, Adachi-ku, Tokyo 120-8551, Japan; 3 Department of Applied Chemistry, School of Engineering, The University of Tokyo, 7-3-1 Hongo, Bunkyo-ku, Tokyo 113-8656, Japan

## Abstract

Direct electric power generation using biological functions have become a research focus due to their low cost and cleanliness. Unlike major approaches using glucose fuels or microbial fuel cells (MFCs), we present a generation method with intrinsically high energy conversion efficiency and generation with arbitrary timing using living electric organs of *Torpedo* (electric rays) which are serially integrated electrocytes converting ATP into electric energy. We developed alternative nervous systems using fluid pressure to stimulate electrocytes by a neurotransmitter, acetylcholine (Ach), and demonstrated electric generation. Maximum voltage and current were 1.5 V and 0.64 mA, respectively, with a duration time of a few seconds. We also demonstrated energy accumulation in a capacitor. The current was far larger than that using general cells other than electrocytes (~pA level). The generation ability was confirmed against repetitive cycles and also after preservation for 1 day. This is the first step toward ATP-based energy harvesting devices.

Development of clean and safe electric power generation methods is a pressing issue. Renewable energy conversions including hydraulic, geothermal or solar generation methods generally depend on the environment. One strategy for stable generation is exploiting biological functions using low-cost fuels^1^. From the viewpoint of directly harvesting electricity, two major methods have been reported: glucose bio-fuel cells incorporating enzyme onto electrodes^2^ and MFCs^3^,^4^ were reported. Differing from these approaches, we have focused on the unique biological function of strongly electric fish. These fish generate large electric pulses using ATP produced from glucose as energy sources to capture prey^5^. We selected the family *Torpedinidae* (electric rays) considering their availability and safety of handling for experiments. As seen in Fig. 1a,b, the ray’s electric organs are aggregated electrocyte columns (500 to 1000/organ) having densely integrated proteins such as ion channels and pumps (molecular motors) on cell membranes of the electrocytes to increase the current. Also, stacked electrocyte layers (~1000/column) increase the voltage^6^. Such complicated and delicate structures are difficult to reproduce artificially by current technology, and are available only in nature, having been developed through evolution.

There have been reports on artificial microdevices incorporating biological functions by cell/tissue engineered approaches including cardiomyocyte actuators^7^,^8^,^9^,^10^, other muscle cell actuators^11^,^12^, vascular systems^13^,^14^, hybrid devices using lung^15^, kidney^16^, and liver^17^. Inspired by these technological developments, we formed the idea to combine electric organ tissue, preserving its structure, and fluidic control systems to create novel functional devices. As seen in Fig. 1c, electric power generation is controlled by simple pressure-driven fluidic control systems through needles inserted into the organs. These needles serve as alternative nervous systems. By using pressure-driven flow, Ach solution can be introduced into small gaps between electrocytes without complicated 3D microstructures.

With respect to energy conversion efficiency, mitochondria-based ATP synthesis efficiency from glucose is about 30% (Supplementary Text 1)^18^. Additionally, ATP consumption efficiency by molecular motors is nearly 100%^19^. Therefore, the total efficiency from glucose to electricity is about 30%. By contrast, glucose fuel cells or MFCs have less than 10% efficiency, because decomposition of glucose to pyruvate provides just 1.5 electrons per glucose molecule while mitochondria synthesize about 30 ATP molecules per glucose molecule and provide 20 electrons by multiple chemical reaction steps^18^. MFCs have more glucose decomposition steps, but their efficiency is still low being around 10%. The ATP-based high energy conversion efficiency from glucose to electricity is significant for large-scale power generation. Another advantage of our concept is that power load following operation (generation with arbitrary timing) is easy by controlling chemical stimuli. Therefore, this concept is also applicable to on-demand small-scale power generation.

To date, several studies have revealed the function of electric organs using structural analysis by electron microscopy^20^,^21^,^22^, chemical analysis by separation techniques^23^,^24^,^25^, and physiological analysis by lipid bilayer membranes^26^. However, analysis of living electric organs or electrocytes have not yet been reported. From an engineering viewpoint, protein-rich membranes of electric organs have been used for bioassay^27^,^28^, but not living whole electric organs. Therefore, investigation of fundamental electrical properties of living electric organs is indispensable.

## Results

### Electric power generation from electric organs by physical stimuli

We first investigated the electric power generation of one member of the *Torpedinidae* family, *Narke japonica*, by physical stimuli to confirm its generation capability. *N. japonica* are often caught in winter in the sea near Japan (Supplementary Text 2). During measurements the head of the electric ray was pressed continuously by hand to make the ray generate electric pulse (Fig. 2a,b). Details of the measurements are described in the Methods section. Short, repetitive pulse type signals were observed for a few minutes (Fig. 2c). The measured peak voltage was about 19 V, and the peak current was about 8 A. These values were roughly comparable to previously reported values although the ray species was different^29^. To show that the generated pulse energy was sufficient to drive a device, LED lighting test was carried out by directly connecting LED to the dorsal and ventral side of the ray. As a result, short time lighting was demonstrated (Supplementary Movies 1 and 2). Therefore, the *N. japonica* used in the experiments were considered to have sufficient electric power generation performance. We also demonstrated energy storage in a capacitor and its usage, showing that the electric power was usable more practically. A circuit with a capacitor (capacitance: *C* = 2500 μF) was constructed as shown in Fig. 2d. The dorsal and ventral sides of the ray were connected to ports 1 and 0 (ground), respectively. The electric energy was accumulated as shown in Fig. 2e. The accumulated voltage (*V*_*c*_) and energy (*E*) were about 2 V and 200 mJ, respectively, as calculated from the equation: *E* = *CV*_*c*_^2^/2. Additionally, LED lighting and toy car driving by accumulated energy in the capacitor were also shown (Supplementary Movies 3 and 4) by connecting ports 2 and 3 after the storage, and the results showed the electric energy can be used for external work. We also applied direct physical stimulation manually by tweezers in an interval of a few seconds when extracting the electric organs with the nerve plexus and central nervous system (Fig. 2f). The organs were extracted from a living ray (Methods and Fig. S1), and it was used soon after extraction. Although the peak height was lower and unstable compared with living ray, similar peak signal was obtained as Fig. 2g. However, the response became weaker by repeated stimulation due to damage to the nerve plexus. In this experiment, we showed that electric organs have large power generation performance and the electric power generation is possible even using extracted electric organs. Simultaneously, however, the difficulty of control by physical stimulation was shown. We concluded that direct stimulation to organs by chemical stimuli is indispensable to realize an electric power generation device.

### Investigation of electric property of electrocytes

We investigated the electric properties of electric organs against chemical stimulation. As seen in Fig. 3a, we used a multi-electrode array system^30^ to confirm that electric organs responded to chemicals. Since there have been no studies with culturing or even temporary storing of electrocytes yet, we tentatively used artificial cerebrospinal fluid (ACSF)^31^ which is commonly used for the measurement of electric properties of muscle cells or neurons. The extracted organs were thinly sliced (about 1 mm thickness) and the slices were temporarily immersed in ACSF solution for survival (Methods). This ACSF solution contained glucose (ingredients to ATP), so that energy was given to the electrocytes. A single slice was then set on an electrode dish, and the electric signals were recorded (Methods) after Ach solution was introduced by both injection and perfusion (Fig. 3a). Figure 3b shows a representative channel for the response of the organs (data for all channels are presented in Fig. S2). In the injection experiment, Ach solution was directly injected into the electrode dish using a micropipette. Ach concentration was 1 mM which is sufficient concentration to get response of membrane proteins^32^. From the result, we confirmed that the slice responded to Ach. The perfusion result showed no physical effect. The obtained voltage was about 100 μV, which is roughly comparable to the outside-cell voltage of acute slice neurons^30^. From these results, survival of electrocytes using ACSF was confirmed for at least 1 h after explanting from the electric ray, and also, electric generation by Ach chemical stimulation was demonstrated. This is the first analysis of living electric organs or cells and it provides fundamental knowledge to design power generation devices. However, this generation method could not effectively utilize the integrated structure of the electrocyte columns. For this purpose, a way to introduce Ach solution while keeping the organ structure is indispensable.

### Demonstration of electric power generation by chemical injection

**Figure 1:**
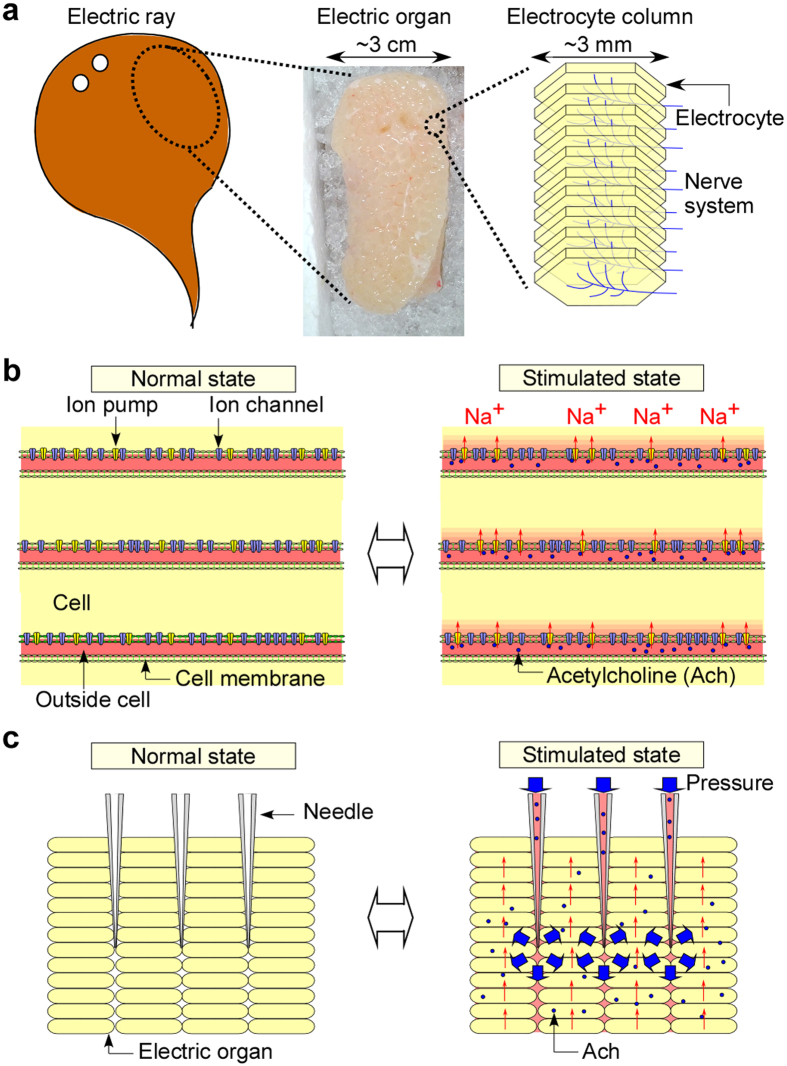
Structure and electric generation principle of the electric organ of *Torpedo* and concept of electric generation by fluid pressure-based chemical stimulation. (**a**) A schematic illustration showing structure of the electric organ and electrocyte columns in it. (**b**) A cross-sectional view of an electrocyte column showing the principle of electric power generation by Ach chemical stimulation from a nerve terminal. In the normal state, ion pumps generate an electric potential difference inside and outside the cells using ATP energy converted from glucose by mitochondria. When Ach stimulates the ion channels, ions are introduced into the cells from outside. This ion flow generates a large current due to the large integration of the proteins on one side of the cell membrane. (**c**) A cross-sectional view showing the concept of electric power generation by the alternative nervous system using fluid pressure. Ach is spread over the organ through syringe needles which are pressure-operated.

We thus developed a method to spread Ach inside the organs by fluid pressure (Fig. 1c) that exploits the sophisticated structure of the electric organs. The experimental setup to inject Ach solution using syringe needles and to measure the electric response is shown in Fig. 4a,b (Methods). We injected 1 mL of Ach solution by manual handing in very short time (within 1 s). We did not employ continuous injection to avoid consuming much amount of chemicals and energy. An electric organ was sandwiched by two pieces of conductive fiber connected to electrodes, and seven stainless steel syringe needles per whole organ were inserted into the organ through the fiber. The voltage and current versus the time of Ach injection were recorded as seen in Fig. 4c. The measured peak voltage was about 91 mV, and the peak current was about 0.25 mA. To show that these peaks were not artifacts due to physical fluctuation by fluid flow, negative control data were obtained. When Ach solution was just added with a micropipette to get a concentration of 1 mM in a dish containing organs already immersed in ACSF without Ach, no clear signals were observed (Fig. 4d). So, injection was shown to be indispensable. If no Ach was present in an injected ACSF solution, there were small signals around the time of injection, probably due to the diffusion of naturally existing Ach in the organs, but the signals were far smaller than for Ach injection (Fig. 4e). Similarly, there were almost no signals (Fig. 4f) when we injected another transmitter 1 μM serotonin solution (a sufficient concentration to stimulate cells^33^) was injected (Fig. 4f). From these experiments, the peak of Fig. 4c was demonstrated to be caused by Ach injection. In the case of using an electric organ that was once frozen at −30 °C in a freezer and then thawed in ACSF solution at room temperature, there were also almost no signals when Ach was injected (Fig. 4g). This was probably because cell membranes and proteins were destroyed by ice forming inside the tissue, which indicated that just living tissue could be used for this power generation.

### Analysis of chemical injection-based electric generation

Following the fundamental proof-of-concept above, we investigated the power generation performance in detail to evaluate this fluid pressure-based generation method. The voltage, current and time duration (half width of peak height) of the organ are compared with those of a living ray in Fig. 5a. The peak voltage and current were smaller compared with the living *N. japonica* probably because not all electrocytes were stimulated by this method. However, the time duration was longer (over 1 s). This was because of the relatively long time for diffusion of Ach through the electric organs by pressure-driven flow, and this long time is very useful for fluidic control of electric power generation. To investigate the repeatability, the voltage and current values were measured versus the number of cycles (about 5 min intervals) at the same conditions as the experiment for Fig. 4c. Figure 5b shows the voltage and the current were decreased, probably because of ATP consumption and organ fatigue. However, the decrease for current was smaller than that for voltage, and it became relatively stable after the second cycle. This was due to the change of the resistance after the Ach injection because the organs conserved the ACSF inside them and that decreased the resistance. The resistance was about 1 kΩ before injection, but became about 100 Ω after it. Therefore, repetitive usability of at least several times was demonstrated. Additionally, an example data showing repetitive (three cycles) current generation about 5 min intervals is shown in Fig. S3. This data indicates that current was generated only just after Ach was injected, and therefore power generation with arbitrary timing was demonstrated. Regarding the usable period, we investigated the voltage and current change using the organs that were kept in ACSF solution for several hours or days. As seen in Fig. 5c, the electric generating function was confirmed to continue for at least 1 day, although both the voltage and current decreased corresponding to elapsed time. This was probably because of a lack of glucose or organ fatigue since the organ storage conditions were not optimized. For long time use, these conditions must be improved. To clarify the effect of the number of needles used, the relationship between the current and needle numbers was investigated in Fig. 5d. We used the same organ and increased the number of needles as 4, 7, 10 and 20, and measured the current every time. We expected that the current would decrease after the second cycle as seen in Fig. 5b; however, the current was linearly proportional to the needle numbers, indicating that the area in contact with Ach increased. Therefore, the current could be controlled by the needle numbers and maximally 0.64 mA was obtained. To design a power generation device, size dependencies of the voltage and current are also important. To compare the voltage and current, whole electric organs and organs finely cut into 3 × 3 cm^2^ and 1 × 1 cm^2^ pieces, including about 100 columns and about 10 columns, respectively, were prepared (also see Fig. S1), and four needles were inserted into each. The electric response for Ach injection of with different sizes (areas) is shown in Fig. 5e. The voltage was not significantly changed even when the size was small. By contrast, current was decreased according to the decrease of the area. Therefore, cutting the organs is effective strategy to obtain a large power generation.

### Creation of electric power generation device using serially connected generation units

Based on the results, we designed a power generation and storage device as shown in Fig. 6a. To accumulate electric power in a condenser, approximately 1 V voltage is necessary. To obtain this large voltage, electric organs were finely cut (3 × 3 cm^2^), packed in the device, and serially connected by conductive wire (Methods and Fig. S4). As alternative nervous systems in this device, stainless steel syringe needles were inserted (four needles/piece of organ). This was one generation unit. Aluminum holders and polydimethylsiloxane (PDMS) sheets sandwiching the organs were required to fix the desired number of needles and to stabilize the signals (especially background fluctuation). For the serial connection and power storage demonstration, we fabricated a prototype device with 16 units as shown in Fig. 6b. Considering setup time of the device (preparing 16 serial units) about 1 h is needed for manual handling, which is actually the time limit to avoid organ deterioration. The maximum voltage 1.5 V was obtained when Ach (1 mM) solution was injected into this device (Fig. 6c). This was roughly 16 times higher than that for 1 layer of tissue (Fig. 4c). The current was 0.22 mA, roughly the same as that for 1 layer. Finally, we accumulated the generated electric power in a capacitor (*C* = 47 μF) using the same, 16 serial unit device (Fig. 6d). Figure 6e confirmed that the accumulated voltage (*V*_*c*_) was about 40 mV with a corresponding voltage loss. Although the accumulated energy was small (*E* = 80 nJ) for actual applications, applicability to general circuit systems was demonstrated. Together with the results in Fig. 5d, the voltage and current were demonstrated to be controlled by serial (unit) or parallel (needle) numbers, respectively. We also fabricated a 1 × 1 cm^2^ organ device (Supplementary Text 3 and Fig. S5) and demonstrated 4 serial unit device, but larger number of serial connections was difficult due to the damage caused by needle insertion.

## Discussion

Here, we discuss the performance of this generator (calculation details are described in Supplementary Text 4). First, we roughly estimated power density at an order level. The power density was calculated as about 10^4^ W/m^2^ for the case of physical stimulation to the living ray, whereas it was 10^−2^ W/m^3^ for the case of fluid-pressure based chemical injection to electric organs. By contrast, representative power densities at room temperature are on the order of 10^0^ to 10^1^ W/m^3^ for glucose fuel cells^34^,^35^,^36^ and 10^−2^ to 10^0^ W/m^2^ for MFCs^37^,^38^,^39^. Therefore, our method is roughly comparable to the MFCs. However, by improving the alternative nervous systems, higher power density up to 10^4^ W/m^2^ can be possibly realized. By integrating the generation units using advanced microfabrication technology, such as employed for fabrication of the 3D microfluidic device^40^ to create more realistic alternative nervous systems, high power would be obtained. The results in this report are positioned as the first step to realize this high power density generator.

From the viewpoint of the membrane protein-based electric power generating system using ATP, single cell level electric power generation detected by the patch clamp method^41^ serves as prior research. Regarding the voltage, both electric organs and the single cell generate about 100 mV although the electrocyte columns of the organs have more than about 1000 serially connected cells^6^. In addition to the difficulty to get simultaneous stimulation of the electrocytes, the difference of the measurement method would be a major reason for the same voltage: the patch clamp method measures voltages inside and outside the cells while our method just measures voltage outside the cells. On the other hand, the current of single cell is about 1 pA order^42^ which is far smaller than the present method (0.1 mA order), simply due to the difference of the covered area: the patch clamp method measures about a micrometer square area while our method measures about a centimeter square area. Even using “whole cell patch clamp” covering a relatively large area, at most 100 pA to 1 nA order^43^,^44^ is the actual limitation. Compared to this, electric organ-based generation is at least six orders higher. Recent progress in microfluidic systems has realized biosensors using isolated membrane proteins coupled with artificial cell membranes (lipid bilayer) fabricated by microfluidics that show controlled volume and performance of power generation, but the current per unit is also about 1 pA order^45^,^46^,^47^, which is similar to the current measured using the patch clamp. Our approach may offer future applications of these bio-hybrid technologies. As simulated previously^48^, our system can be a model for electric power generation systems using a biological generation method based on the ATP energy conversion system if electrocytes, electric organs or similar systems can be artificially created.

## Methods

### Measurement of voltage and current from *N. japonica*

**Figure 2:**
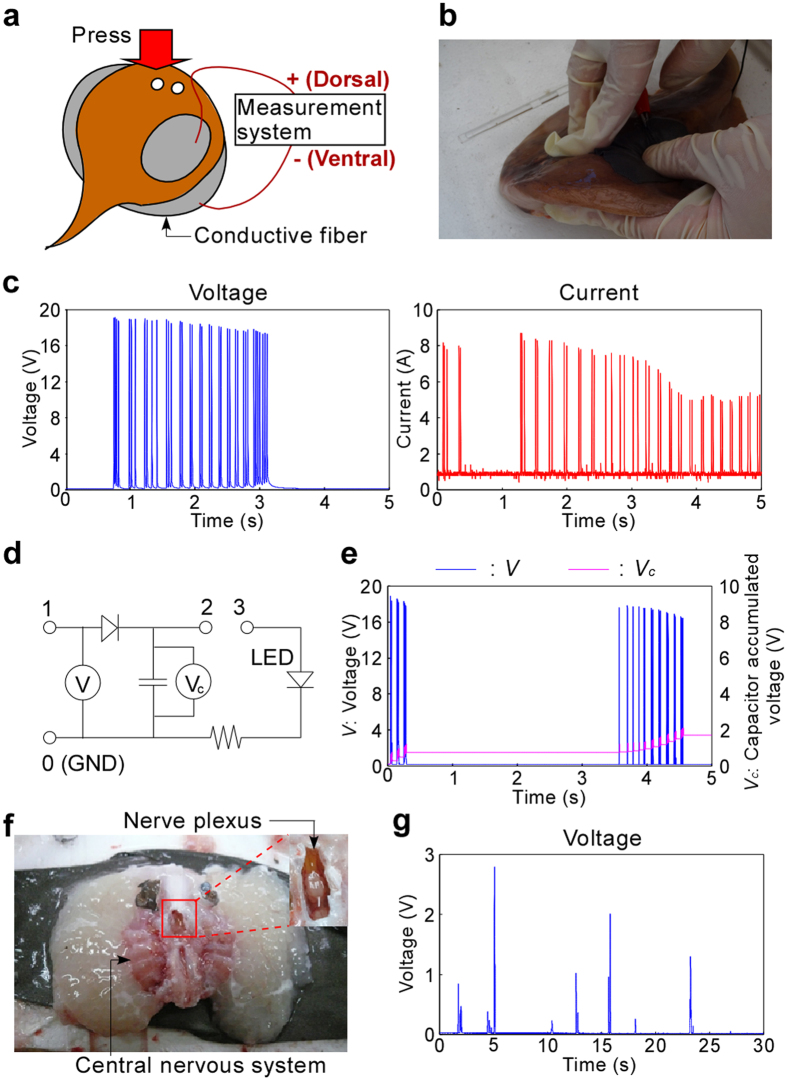
Measurement of electric power generation from electric organs by physical stimuli. (**a**) Illustration showing the method to measure voltage and current generated by electric organs. (**b**) Photo showing the head of the electric ray (*N. japonica*) being continuously pressed by hand during the measurement. (**c**) Time course for measured by physical stimuli for 30 s. (**d**) A circuit for capacitor storage and LED lighting. (**e**) Time course for measured voltage of the electric ray and corresponding voltage accumulated in a capacitor. (**f**) Photos showing a pair of extracted electric organs with the central nervous system and nerve plexus. (**g**) Time course for measured voltage when applying direct physical stimulation to the nerve plexus using tweezers.

To investigate the electric property of *N. japonica* itself, we measured its generation property. We used freshly caught rays, and carried out the experiments near a fishing port. The rays were obtained from Aquamarines (a fishing company located in Minamiise, Mie, Japan). We used two pieces of conductive fiber (DEV-10055, SparkFun Electronics, Niwot, USA) that were pasted onto a ray as shown in Fig. 2A. To make the ray generate electricity, its head was pressed by a hand. To measure and record the voltage, a data logger was used (NR-2000, Keyence, Osaka, Japan) with an amplifier connected to the electrodes by interfaced software (WAVE SHOT! 2000, Keyence). To measure the current, a shunt resistance (100 Ω) was used. The time resolution was 1 ms. An analog circuit was made on a bread board with a capacitor (Akizukidenshi, Tokyo, Japan).

### Explant method of electric organs

The procedure to obtain the electric organs is described here (also see Fig. S1). To explant a pair of electric organs, the electric ray was cooled for about 10 min on ice to anesthetize it. Then, the skin on the electric organs was detached using scissors from the dorsal side and a pair of tweezers was used to pull the skin away. After revealing the organs, they were explanted by scissors along the edge of the organ. Skin on the ventral side was not detached from the organ to allow identification of the direction (dorsal and ventral) of the organ.

### Treatment of electric organs

Explanted electric organs were immersed in ACSF for fish which is physiological saline with glucose. Specifically, ACSF for angler^49^ was used. The solution contained 205 mM of sodium chloride, 8 mM of potassium chloride, 3 mM of magnesium sulfate, 2.2 mM of calcium chloride, 2.2 mM of sodium hydrogen carbonate, 0.5 mM of potassium dihydrogen phosphate, and 10 mM of glucose. These reagents were purchased from Sigma-Aldrich (St. Louis, USA). A whole organ could be contained in a 200 mL beaker, and the organ was used within 1 h of explanting. After removing the organ from the ACSF solution, it was quickly used for the experiments.

### Measurement system using multi-electrode array

For measurement, a commercial 64-channel multisite recording system (MED64, Panasonic Alpha-Med Sciences, Ibaraki, Japan) was used for extracellular field potential recordings. Fundamental procedures for the preparation of the MED were almost the same as described previously^50^. Acute slices of electric organs were prepared from ACSF-immersed electric organs. Coronal slices (1 mm diameter) was trimmed using a biopsy punch, and also sliced to be about 0.5 mm thick by scissors. The slices were transferred to a chamber with oxygenated ACSF at room temperature. The slices were allowed to equilibrate for 1 h before electrophysiological recording began. The size of each electrode in the array was 50 × 50 μm, and all 64 electrodes were arranged in an 8 × 8 square pattern with an inter-electrode distance of 300 μm. The probe surface was rinsed 3 to 5 times with sterile distilled water immediately before use in each experiment.

For chemical stimulation, a well-known neurotransmitter, Ach (acetylcholine chloride) which was purchased from Wako Pure Chemical Industries (Osaka, Japan) was used. It was dissolved in ACSF (1 mM of acetylcholine chloride).

The slice was continuously perfused with oxygenated, fresh ACSF at a rate of 2 mL/min with the aid of a peristaltic pump (PERI-STARTM, WPI, Sarasota, USA). For the injection experiments, Ach solution was directly added into the measurement chamber using a micropipette. For the perfusion experiments, ACSF solution was replaced by Ach solution, and it was continuously circulated by the pump until the solution reached the measurement chamber.

### Method of electric organ stimulation and electric signal recording

**Figure 3:**
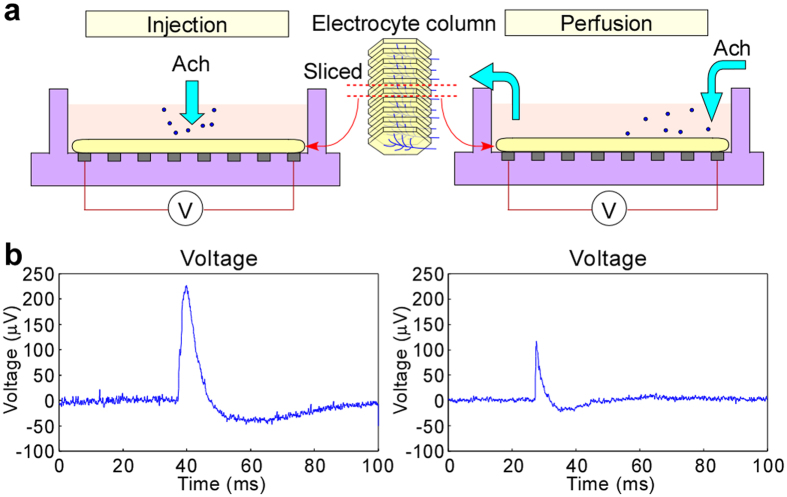
Measurement of electric property of electrocytes using the MED 64 system. (**a**) Cross-sectional schematic views showing the method to measure voltage of the sliced electric organ for Ach stimulation by injection and perfusion conditions. (**b**) Time courses for measured voltage of representative electrodes corresponding to the two conditions.

**Figure 4:**
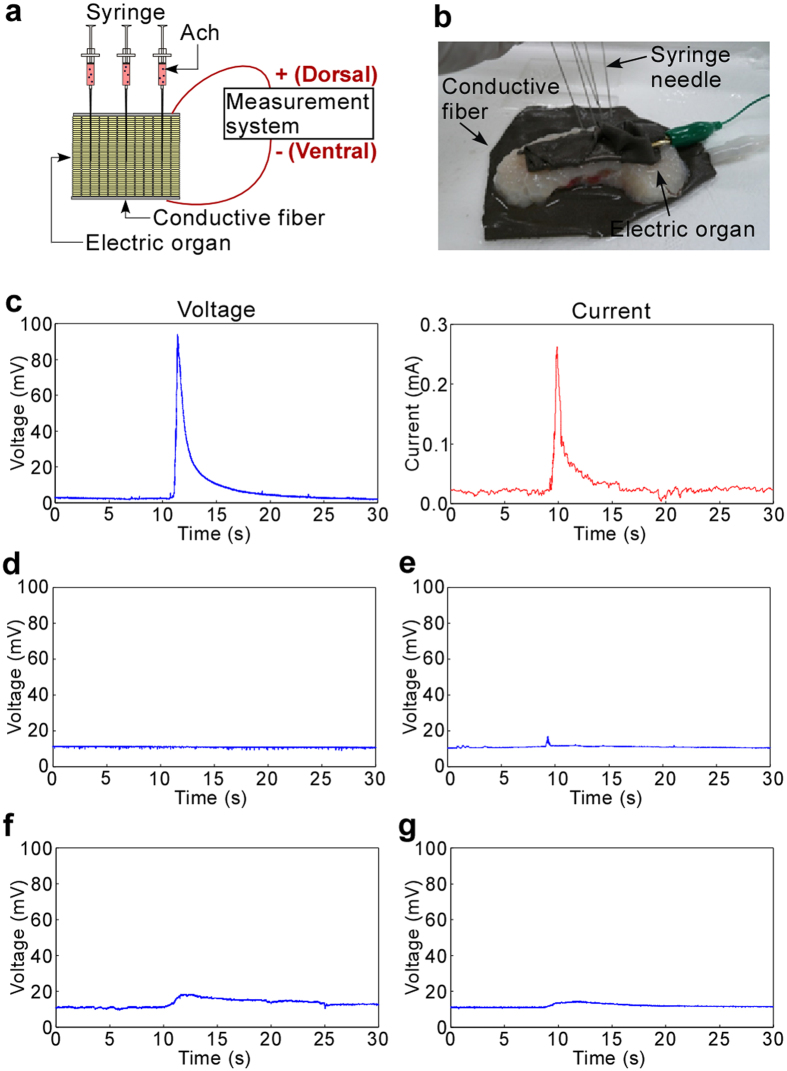
Electric power generation demonstration by chemical injection. (**a**) A cross-sectional schematic illustration showing the method to measure voltage and current generated by the electric organ. (**b**) Photo of the electric organ with syringe needles and conductive fiber. (**c**) Time course for measured voltage and current generated by the chemical stimulation for 30 s. The injection timing was around 10 s. (These conditions also apply to Figs 4d–g and 5c,e.) (**d**) Time course for measured voltage of a case just adding Ach solution to an electric organ without using the needles inserted into it. (**e**) A case injecting just ACSF (no Ach). (**f**) A case injecting serotonin instead of Ach solution. (**g**) A case using a once frozen and thawed organ.

**Figure 5:**
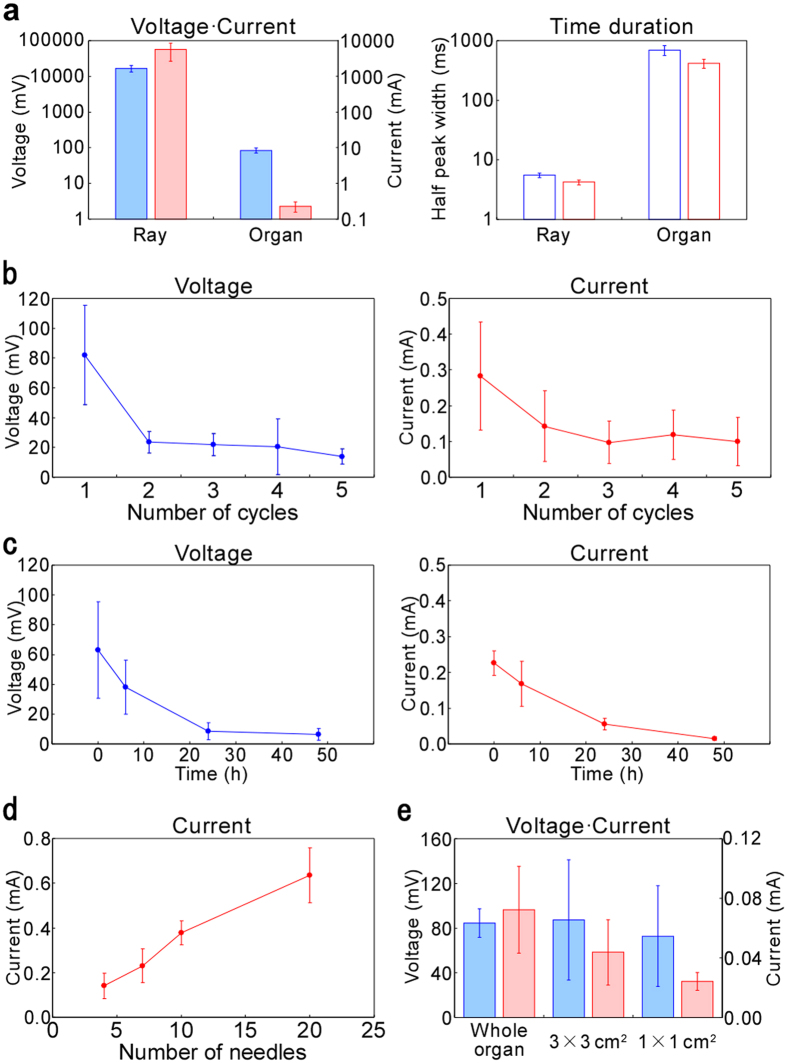
Detailed investigation and analysis of chemical injection-based electric generation. (**a**) Comparison of voltage (solid blue bar), current (solid red bar) and time duration (voltage: open blue bar, current: open red bar) indicated by their half width peak heights against physical stimulation to *N. japonica* (Fig. 2c) and chemical stimulation to the electric organ (Fig. 4c). (**b**) Measured voltage and current versus number of cycles. (**c**) Measured voltage and current versus the time electric organs were kept in ACSF solution at room temperature. (**d**) Measured current versus number of needles. (**e**) Measured voltage (solid blue bar) and current (solid red bar) versus size of electric organs (whole or pieces). All error bars indicate ± S.D.; n = 3 in this figure.

**Figure 6:**
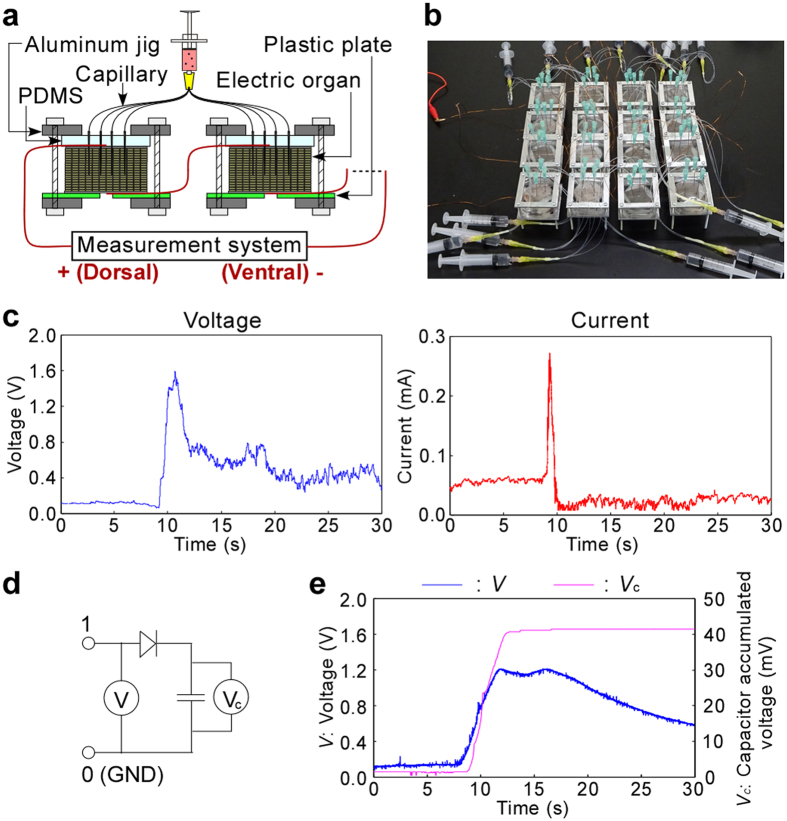
Electric power generation device using serially connected generation units. (**a**) A cross-sectional schematic illustration showing the method to measure voltage and current generated by serially connected electric organs. (**b**) Photo of the device connecting 16 generation units. (**c**) Time course for measured voltage and current using the 16 serial units for 30 s. Injection timing is around 10 s. (**d**) A circuit for capacitor storage. (**e**) Time course for measured voltage of the 16 serial units and corresponding voltage accumulated in a capacitor.

For power generation using electric organs experiments by chemical injection, Ach solution was manually injected into the organs (1 mL) using 21 gauge needles (Terumo) with 1 mL syringes (Terumo). The electric organ was sandwiched by two pieces of conductive fiber connected to electrodes as shown in Fig. 3A, and stainless steel syringe needles were inserted into the organ through the fiber. The voltage and current measurement and recording system are similar to that for the living ray itself. The resistance was measured by a digital multimeter (P-16, METEX, Seoul, Korea). To measure the small current, a current-input preamplifier (LI-76, NF Corporation, Yokohama, Japan) (gain: 10^4^ V/A) was used between the electrodes and data logger.

### Device fabrication

A power generation device incorporating small pieces of electric organs was fabricated as follows (also see Fig. S4). The device mainly consisted of aluminum jigs, polydimethylsiloxane (PDMS) layers and polyvinylchloride (PVC) layers. The aluminum jig and PVC layers were fabricated using a milling machine to be 6 × 6 cm^2^. The PDMS and PVC layers with a piece of the electric organs were sandwiched by the jigs using four screws (3 mm diameter), one on each of the four corners. A PDMS elastomeric sheet was prepared by casting. PDMS prepolymer (Silpot 184 W/C, Dow Corning Asia, Tokyo, Japan) was prepared by mixing PDMS base with a curing agent in a 10:1 ratio by weight, poured on a plastic case to be about 5 mm in thickness, degassed and cured at 70 °C for 3 h, peeled and cut to be a size of 6 × 6 cm^2^. Perfluoroalkoxyalkane (PFA) capillaries made of (0.5 mm I.D, 0.7 mm O.D., EXLON-Tubing, IWASE, Yamato, Japan) were bundled (four capillaries per unit) and inserted into an edge-cut pipette chip (110NEW, Quality Scientific Plastics, San Diego, USA). They were fixed with epoxy glue (Araldite, HUNTSMAN JAPAN, Kobe, Japan), and the pipette chip was connected to a 26 gauge needle (Terumo, Tokyo, Japan) also by the epoxy glue. The needle was connected to a 5 mL syringe (Terumo) which is pressed by hand for injection. The other end of each of the PFA capillaries was inserted into a 21 gauge needle and fixed with the epoxy glue, one by one.

### Ethical approval

All animal experiments were approved by the Animal Care and Experimentation Committee of RIKEN and were carried out in accordance with the approved guidelines.

## Additional Information

**How to cite this article**: Tanaka, Y. *et al.* An electric generator using living *Torpedo* electric organs controlled by fluid pressure-based alternative nervous systems. *Sci. Rep.* **6**, 25899; doi: 10.1038/srep25899 (2016).

## Supplementary Material

Supplementary Information

Supplementary Movie 1

Supplementary Movie 2

Supplementary Movie 3

Supplementary Movie 4
