# Supplementary material for: An electric generator using living Torpedo electric organs controlled by fluid pressure-based alternative nervous systems
Source: Sci Rep. 2016 May 31;6:25899. doi: 10.1038/srep25899 (PMC4886531; doi:10.1038/srep25899)

**Supplementary Information**

# An electric generator using living *Torpedo* electric organs controlled by fluid pressure-based alternative nervous systems

**Yo Tanaka,*^1^ Shun-ichi Funano,^1^ Yohei Nishizawa,^1,2^ Norihiro Kamamichi,^2^ Masahiro Nishinaka^3^ and Takehiko Kitamori^3^**

^1^ Quantitative Biology Center (QBiC), RIKEN, 1-3 Yamadaoka, Suita, Osaka 565-0871, Japan

^2^ Department of Robotics and Mechatronics, Tokyo Denki University, 5 Senju-asahi-cho, Adachi-ku, Tokyo 120-8551, Japan

^3^ Department of Applied Chemistry, School of Engineering, The University of Tokyo, 7-3-1 Hongo, Bunkyo-ku, Tokyo 113-8656, Japan

*****To whom correspondence should be addressed: E-mail: yo.tanaka@riken.jp.

TEL: +81-6-6105-5132, FAX: +81-6-6105-5241

**Supplementary Text 1**

**- Energy conversion efficiency -**

Theoretical energy efficiency was calculated as follows. The reaction of ATP is presented as the following equation.

ATP + H_2_O = ADP + Pi + 7.3 kcal/mol

If glucose is burned completely, the reaction is presented as the following equation.

C_6_H_12_O_6_ + 6O_2_ + 6H_2_O = 6CO_2_ +12H_2_O + 720 kcal/mol

Therefore, if 30 ATP molecules are produced from one glucose molecule, the efficiency was calculated as 30×7.3/720 = 30%. By contrast, glucose decomposition to pyruvate by enzyme just produces only 2 ATP molecules. This corresponds to about 2% energy efficiency. Depending on the methods (kinds of enzymes or bacteria), the step number is different. However, equivalent ATP synthesis efficiency to mitochondria performance has not yet been realized, and therefore the actual limit is approximately less than 10% efficiency.

**Supplementary Text 2**

**- Situation of *N. japonica* fishing -**

To carry out experiments stably, *N. japonica* must be provided constantly. However, their availability depends on the season. Therefore, we investigated the numbers of *N. japonica* caught in each season and summarized the findings in Table S1. The numbers of *N. japonica* brought to Nayaura fishing port in Minami-isecho, Mie Prefecture, Japan were counted during a one month period each season. Usually, electric rays are caught with other fish, fishermen do not aim at catching just electric rays. About 50 fishing boats per day come to the port. Although the fluctuation is very large, some electric rays are caught almost every day in winter and spring. By contrast, the number is small and unstable in summer and early fall. One of the reason for the low numbers is the sea temperature. When the sea temperature is low, the electric rays come into shallow water, but they go out to deep water when the shallow sea temperature is high. Another reason is caused by the fishing method. During winter and spring, both fixed net and gill net methods are adopted. Conversely, only fixed nets which catch fewer electric rays than gill nets are adopted during summer and autumn. In summer and autumn as well, the electric charge of the electric rays is very weak and unsuitable for experiments. Therefore, we focused on carrying out experiments in winter and spring.

**Supplementary Text 3**

**- Miniature device demonstration -**

In order to investigate how much we can miniaturize the power generation device, we fabricated and demonstrated a miniaturized device using 1×1 cm^2^ cut organs. As seen in Fig. S5, the fundamental structure was similar to the 3×3 cm^2^ organ device. However, to densely arrange the needles, their tips were cut by a leutor and the needles were fixed in a plastic plate. Once Ach solution was pooled between two plastic plates, it was injected into the organ through the needles. By using four serial generation units, maximally about 400 mV was obtained which was roughly four times higher than the voltage for the single layer generation (Fig. 4c).

**Supplementary Text 4**

**- Calculation of power density -**

Here, we discuss details of the calculation steps for power density and the theoretical energy conversion efficiency for the present method (*N. japonica* and electric organs) and previously reported methods (glucose decomposition by electrodes and microorganisms).

Power density was calculated as follows. The area of the *N. japonica* used for this experiment was about 150 cm^2^. From the voltage and current shown in Fig. 2c (19 V, 8 A), the power density was calculated as 1.0×10^4^ W/m^2^. Electric organ-based performance was assumed according to Fig. 5c (1.5 V, 0.2 mA). The area was calculated as 3×3 cm^2^/organ multiplied by the serially connected number of 16. The calculated value was 2.0×10^-2^ W/m^2^.

**Supplementary Table, Figure and Movie legends**

**Table S1 |** Number of *N. japonica* brought to the Nayaura fishing port. For each season, the number was counted on every fishing day for about 1 month.

**Figure S1 |** Photos showing the procedure to extract electric organs. (a) Cutting skin on the dorsal side. (b) Detaching skin around the dorsal area. (c) Exposed nerve terminals and electric organs. (d) Extraction of electric organs. e, Extracted electric organs (Left: Dorsal side looks upward, Right: Ventral side with skin looks upward). f, Electric organs immersed in ACSF solution. g, Photos of whole and cut electric organs with a 1 yen coin (2 mm diameter) for size comparison.

**Figure S2 |** Electric response of sliced electric organs obtained using the multi-electrode array system. These graphs show data of 64 channels corresponding to Fig. 3b. Channels 57 in the injection condition and 64 in the perfusion condition) are presented in Fig. 3b. (Channels 57 and 64 had the maximum values among the 64 channels. All data were expanded in the horizontal axis direction). The difference in signal intensity in each channel indicates that the response was not an artifact or noise from outside.

**Figure S3 |** Time course for measured current for 15 min showing the response to repetitive (three cycles) Ach injection at 5 min intervals.

**Figure S4 |** Supplemental explanation of an electric power generator using electric organs by fluid pressure-based chemical stimulation (Fig. 5). (a) Design of each layer of the device. Inset: Cross-sectional view of a single generation unit along the dotted blue line. (b) Photo of components of the device (aluminum jig, PDMS sheet and plastic plate). (c) Photos of a constructed device (top and side views). (d) Procedure to introduce electric organs into the device.

**Figure S5 |** Miniaturized version of a power generator using electric organs by chemical injection. (a) Design of each layer of the device. Inset: Cross-sectional view of a single generation unit along the dotted blue line. (b) Photos of the device. (c) Time course for measured voltage and current for 30 s.

**Movie 1 |** This movie shows real-time direct LED lighting from generated electrical pulse of *N. japonica* by direct physical stimulation by a hand applying pressure corresponding to Fig. 2.

**Movie 2 |** This movie is a slow motion version of Movie 1 (1/4 speed).

**Movie 3 |** This movie shows real-time LED lighting for about 10 s using accumulated energy in a capacitor from *N. japonica* by direct physical stimulation by a hand applying pressure corresponding to Fig. 2. After connecting port 0 to the ventral side and port 1 to the dorsal side to accumulate energy, ports 2 and 3 were connected to the LED (see Fig. 3d).

**Movie 4 |** This movie shows real-time toy car running by using the energy accumulated in a capacitor from an electric ray by direct physical stimulation by a hand applying pressure corresponding to Fig. 2. The operation to accumulate and use energy is similar to that for LED lighting.

**Table S1**

| **Day** | **1** | **2** | **3** | **4** | **5** | **6** | **7** | **8** | **9** | **10** | **11** | **12** | **13** | **14** | **15** | **16** | **17** | **18** | **19** | **20** | **21** | **22** | **23** | **Total** |
| --- | --- | --- | --- | --- | --- | --- | --- | --- | --- | --- | --- | --- | --- | --- | --- | --- | --- | --- | --- | --- | --- | --- | --- | --- |
| Winter  (Jan.18-  Feb.16) | 2 | 3 | 5 | 1 | 0 | 4 | 5 | 0 | 1 | 1 | 0 | 3 | 6 | 14 | 19 | 9 | 7 | 0 | 26 | 26 | 18 | 0 | 6 | 156 |
| Spring  (Apr.16-  May.22) | 25 | 14 | 5 | 28 | 22 | 0 | 0 | 0 | 11 | 7 | 7 | 12 | 1 | 7 | 12 | 18 | 3 | 2 | 33 | 34 | 31 | 48 | 25 | 345 |
| Summer  (Jul.18-  Aug.16) | 6 | 0 | 1 | 0 | 2 | 0 | 1 | 0 | 1 | 3 | 0 | 0 | 0 | 3 | 1 | 0 | 0 | 5 | 3 | 3 | 0 | 1 | 0 | 30 |
| Fall  (Oct.17-  Nov.16) | 1 | 0 | 0 | 0 | 0 | 2 | 1 | 5 | 0 | 2 | 0 | 1 | 0 | 22 | 14 | 39 | 49 | 38 | 26 | 25 | 42 | 41 | 38 | 346 |

**Figure S1**


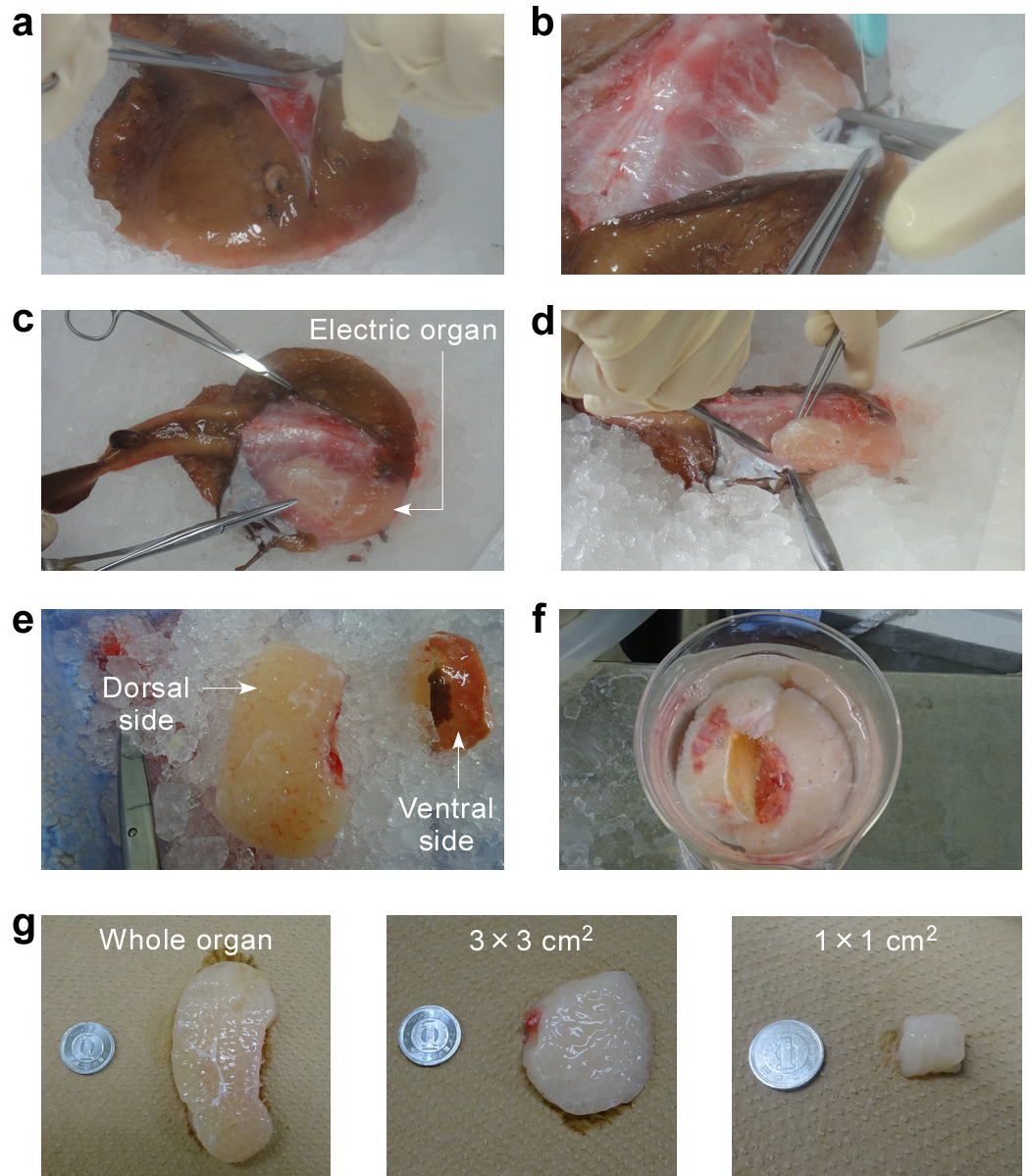


**Figure S2**


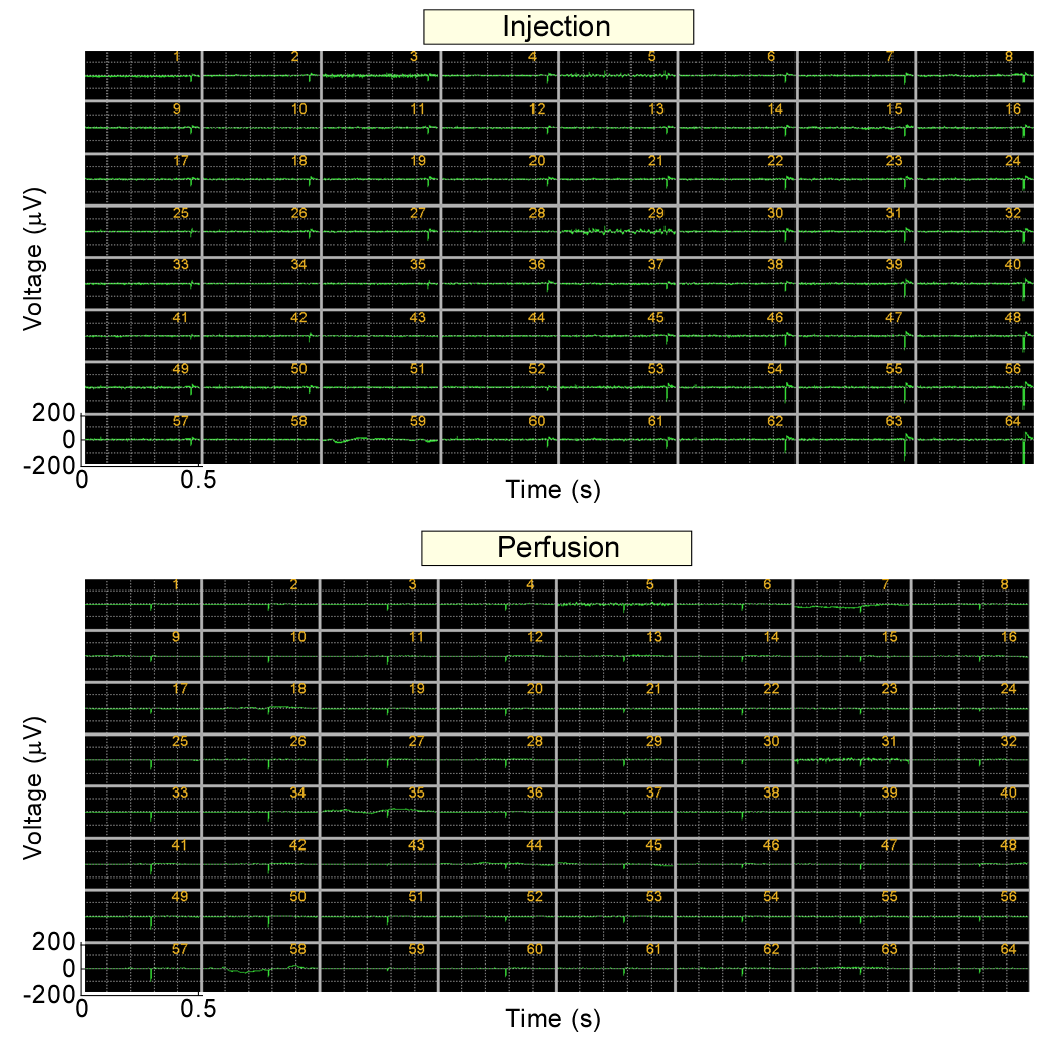


**Figure S3**


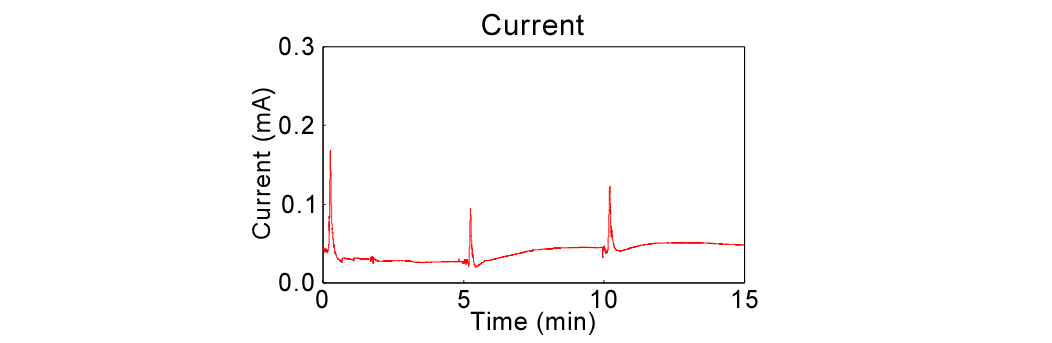


**Figure S4**


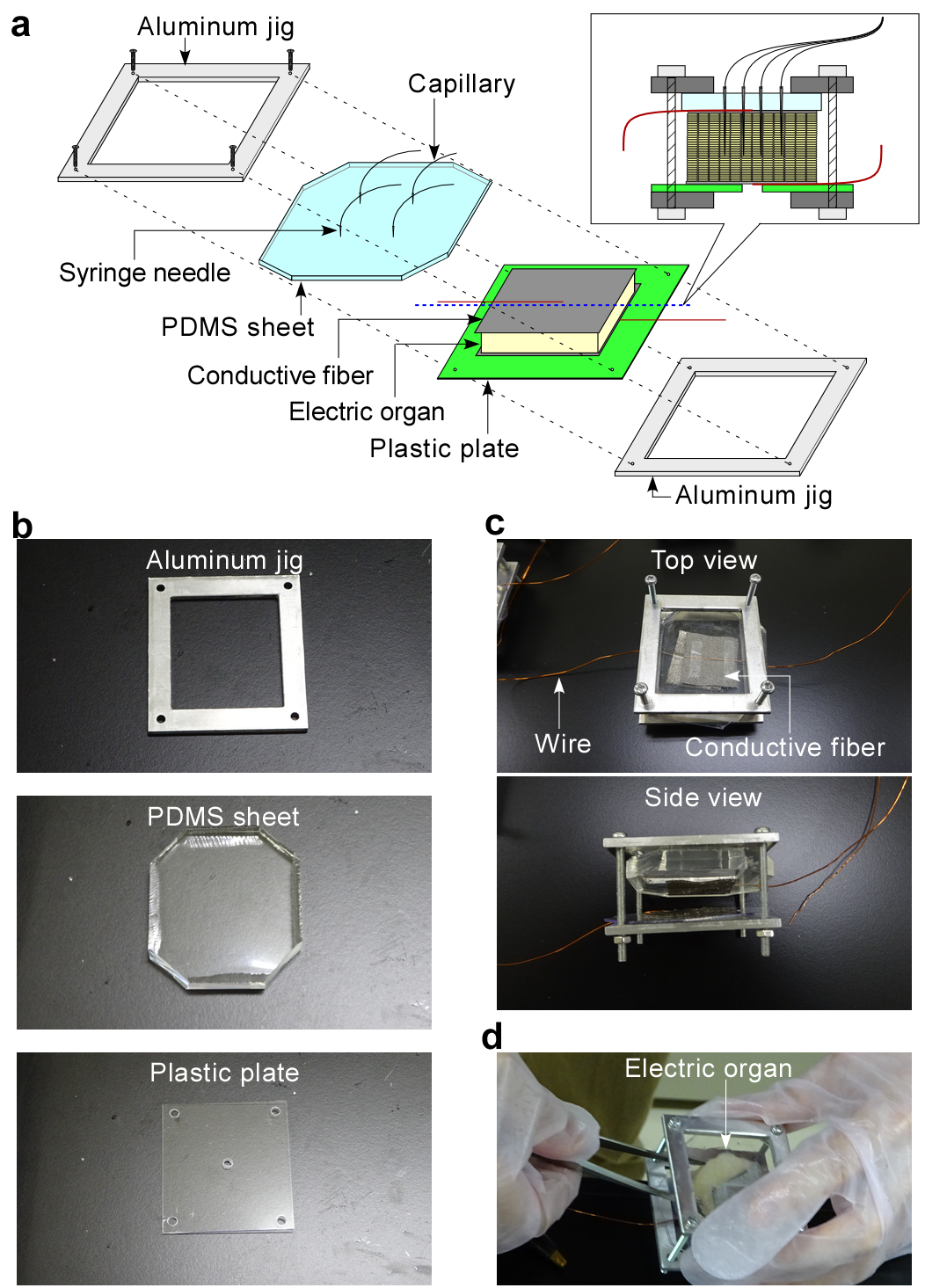


**Figure S5**


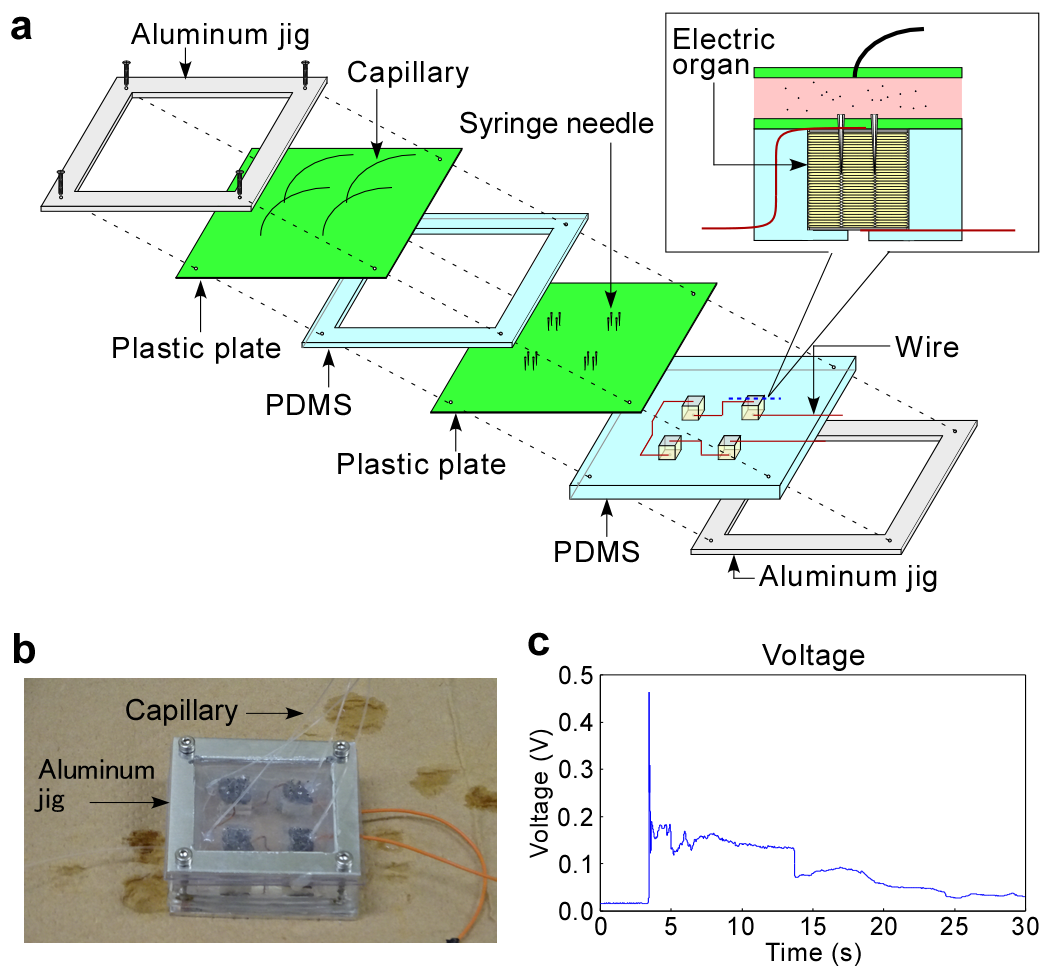

Supplement: Supplementary Information [file srep25899-s1.doc]
